# Epidemiology of horse trauma: a literature review

**DOI:** 10.1007/s00068-023-02436-0

**Published:** 2024-01-24

**Authors:** Emily K. Neville, Henry Hicks, Christine C. Neville

**Affiliations:** 1 School of Medicine, Wagga Wagga Rural Clinical School, University of Notre Dame Australia, Wagga Wagga, New South Wales 2650 Australia; 2 Department of General Surgery, Wagga Wagga Base Hospital, Wagga Wagga, New South Wales 2650 Australia; 3 School of Nursing and Midwifery, University of Southern Queensland, Toowoomba, QLD 4350 Australia

**Keywords:** Horse, Riding, Trauma, Epidemiology, Injuries, Review

## Abstract

**Purpose:**

Horses are used for many recreational and occupational activities. They are large, strong, temperamental, and unpredictable animals and people involved with them are at risk for injuries, from minor abrasions to severe injuries that may lead to death. This review reports on horse trauma in relation to the characteristics of injured equestrians, characteristics of horse trauma, and clinical outcomes.

**Methods:**

A literature search was conducted from health-related electronic databases to identify studies from 2018 to 2023. The search returned 115 relevant full-text articles but after screening and assessment for eligibility, 39 were included in this review for a detailed examination of horse trauma epidemiology. Most studies were undertaken in the USA and the most used method was a retrospective review of hospital or trauma registry data.

**Results:**

There have only been very slight changes in horse trauma numbers and outcomes over the past 5 years. Most injuries often follow falls and kicks. Females in their late-20 s to mid-30 s who are recreational equestrians are the group most represented in the data. The commonest injuries include fractures, and head, thoracic, and abdominal trauma. Most individuals with horse trauma were treated in the Emergency Department and discharged. For the equestrians who were admitted to hospital, around one-third required surgery. Mortality rates are very low.

**Conclusion:**

The popularity of occupational and recreational horse activities does not seem to wane and horse trauma continues to represent a significant concern for the health system. Health care workers need to be cognizant of the scope of trauma presentations as the mechanisms of injury can be complicated putting the equestrian at a high risk of associated injuries that may be life-threatening.

## Introduction

Horses are large, heavy, and unpredictable animals that run at high speeds and can kick, strike, and bite. Despite these inherent risks, horses and humans have close ties in occupational and recreational activities with all age and gender groups involved. In the United States of America (USA), it is estimated that each year 20 million people aged 16 years and older participate in horse-related activities [1]. British Equestrian reported 1.8 million regular equestrians and approximately 1% (283,000) of the Australian population are horse enthusiasts [2, 3]. Most horse trauma occurs during recreational activities, with a greater representation of adult females, children, and teenagers. Injuries occur in both mounted and unmounted situations with many injuries considered as serious. While fractures are the predominant injury sustained, it is abdominal trauma and head injuries, ranging from concussion through to severe structural brain injury, that are usually responsible for longer hospitalization and death.

Horse trauma incurs significant costs at several levels. In a Swedish study (1997–2014), Meredith et al. [4] gave a conservative calculation of 3.2 million Euro annually of 1800 Euro per injury event. A USA study which analyzed thoroughbred horse farm workers’ compensation insurance claims (2008–2015) found that the total amount paid on claims were USD $11,181,268 and the total number of lost-time days was 18,412 [5]. When analyzing the costs for treatment at a USA Level 1 Trauma Centre for the period of 2010–2013, Adler et al. [6] calculated the mean expenditure per injured patient at USD $29,737 with the total expenditure for all the patients (*N* = 222) more than USD $6.5 million. Jones et al. [7] estimated the cost for 332 patients in New Zealand (2012–2016) at NZ $2.6 million with the costliest 10% of patients accounting for 36.9% of the overall cost (NZ $929,285). The average cost per patient was NZ $7805 with males costing more (NZ $8901) than females (NZ $7391). Adults were more expensive to treat than children (0–14 years of age) and over 76.3% of the total cost was for recreational equestrians.

Four previous literature reviews on horse trauma were sourced [8–11]. The focus of Zuckerman et al. [10] was specifically on traumatic brain injury and Gates and Lin [11] on head and spinal injuries. Havlik [9] and Meredith et al. [8] took a broader review of injuries with Havlik [9] reviewing literature from 2007 to 2009 and Meredith et al. [8] reviewing literature from 1973 to 2017. Therefore, it is timely to provide an updated review of the literature. The epidemiology of horse trauma is difficult to specifically characterize with some studies analyzing large national databases through to small, single institutions from a variety of countries and geographical areas. Taking this diversity into account, this review was designed to critically appraise and summarize reports on horse trauma as well as examine the characteristics of injured equestrians, characteristics of horse trauma, and clinical outcomes.

## Methods

**Fig. 1:**
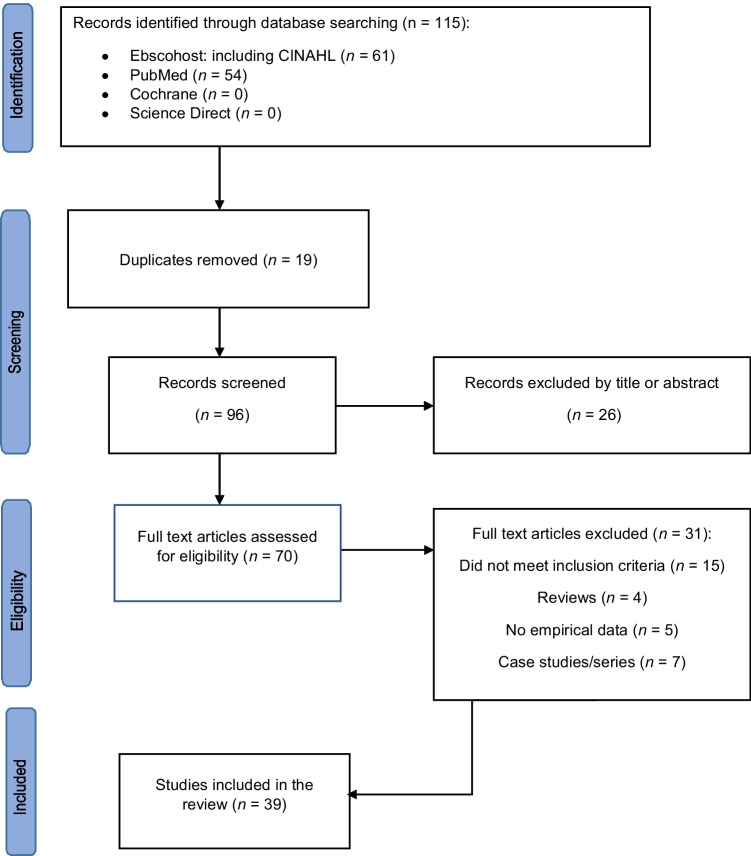
Study selection process

Search terms used were “horse/hors* or equine or equestrian and trauma or injury/injur*or accident/accident* or fall” to identify English language studies in academic journals from 1 Jan 2018 to 30 June 2023. See Fig. 1 for details of the study selection process.

## Results

There were 39 articles which detailed the injury epidemiology of horse trauma. Most studies (30.8%) were published in 2019. Over 30% (*n* = 13) of the studies were conducted in the USA, followed by Australia (*n* = 5), Ireland (*n* = 4), and then various other countries including the UK, Israel, and New Zealand. There was one international study, 12 that analyzed national databases, eight that analyzed data from multiple centers, and 18 that reported on single centers. Retrospective analysis of medical records was the most common method (87.2%; *n* = 34), then cross-sectional (7.7%; *n* = 3) with two prospective studies (5.1%). Three studies specifically examined pediatric samples. Most studies (26/39) reported on trauma in all horse-related activities, whereas five investigated injuries in horse racing, three looked at injuries on farms, three examined injuries from horse sport competitions, and there were individual studies for amateur, collegiate, and riding school equestrians. The majority (29/39) of studies reported on the overall injury pattern to the entire body, while four detailed maxillofacial injuries, three specifically examined head and neck trauma, two concentrated on pelvic injuries, and one looked a radial neck in children. More summary details are provided in table format (see Table 1).

**Table 1:** Summary data for 39 epidemiological studies of horse trauma (2018–2023)

Country, year [reference]	Study design (time)	*N*	Mean age (SD) Median age (IQR)	Gender % F/M	Style of horse activity	Key findings
International studies						
Canada, Nth Ireland, UK, USA, Australia (2021) [12]	Cross -sectional	511	39.42 (14.08)	96.3/6.2	Eventing, show jumping, dressage, Western, endurance, trail	• Relationship between concussion, depression, and low well-being • Mental health support needed
National studies						
Portugal (2023) [13]	Retrospective (2 months)	216	18 (IQR 10–53)	48.6/51.4	Riding schools	• Equestrians ≤ 1 year of experience • Most equestrians have at least one injury
USA (2022) [14]	Retrospective (1 year)	3911	Occupational injuries: predominantly males aged 18–49 years Recreational injuries: predominantly females aged < 18 years and > 60 years		All activities	• Occupational injuries likely to upper limbs of working age, minority males • Recreational injuries likely to head of younger and older females
UK (2022) [15]	Retrospective (1 year)	352	34.22	155/37	Horse racing	• Most staff sought time-off for fractures, but < half sought time-off for concussion
USA (2021) [16]	Retrospective (10 years)	24,791	46.85 (15.33)	49.47/50.5	All activities	• Commonest injury was thorax, but head and neck injuries had the highest mortality • Hospital admission from horse trauma ˃ football, motor vehicle racing, and skiing
Ireland (2021) [17]	Cross-sectional (1 year)	92	NA	5/87	Jockeys (off track)	• Non-racing injuries can affect jockeys’ ability to work
USA (2021) [18]	Retrospective (1 year)	5731	42 (21)	57/43	All activities	• 86% was horse trauma • Severe injury patterns like motor vehicle collision patients
USA (2020) [19]	Retrospective (27 years)	1,836,536	29.8 (IQR, 15.5–44.1)	63.7/36.3	All activities	• Decline in horse trauma, but still common • Clinicians should be aware of the spectrum of presentations
Sweden (2019) [4]	Retrospective (19 years)	29, 850	26.8 (16.1)	89.6/10.4	All activities	• Decrease in fatalities but head injury still high • Type of injury changes with age group
New Zealand (2019) [20]	Prospective (7 years)	406	NA	NA	On farms	• Horse riding was the MOI with the highest number of total events, with 91.6% occurring during non-farming activities
USA (2019) [1]	Retrospective (5 years)	21,899	NA	68.5/31.5	All activities	• Most injuries (85.9%) were treated and released from the ED with females aged 15–19 the most prevalent • Need for injury prevention programs
New Zealand (2018) [7]	Retrospective (5 years)	701	36.5 (19)	69.2/30.8	All activities	• Severity of horse trauma is less than reported • The volumes and costs of injury remain a burden
Ireland (2018) [21]	Retrospective (9 years)	494 ± 92.6	NA	NA	Horse racing	• Injuries reduced over 9 years • Ways to reduce fall risk in point-to-point racing needed as serious injuries commoner than professional racing
Multicenter studies						
USA (2022) [22]	Retrospective (20 years)	6730	27.80 (range 2–91)	71.9/28.1	All activities	• When the severest craniomaxillofacial injury was fracture, the chance of hospital admission was greater than when the severest injury was contusion
USA (2022) [23]	Retrospective (5 years)	443, 730	≤ 17 years: 33.47% ≥ 18 years: 66.53%	91.51/8.48	Competition, e.g., dressage, hunter-jumper, eventing	• Equestrian sports have an overall injury rate of 780 per 100,000 athlete exposures • Hunter–jumper and eventing have more injuries than non-jumping disciplines
France (2020) [24]	Retrospective (12 years)	18	9.17 (2.45)	94/6	All activities	• Radial neck fractures in children were more severe than other MOIs
USA (2020) [25]	Cross-sectional	73	20.3 (1.90)	97.3/2.7	Collegiate equestrians	• Concussion is common • Upper extremity injury is associated with concussion risk
Australia (2020) [26]	Retrospective (25 years)	8	47 (8–73)	37.5/62.5	All activities except horse racing	• Lethal head injuries often follow falls and kicks • Traumatic lesions at autopsy include fractures, and blunt craniocerebral, thoracic, and abdominal trauma
USA (2020) [5]	Retrospective (8 years)	2276	≤ 30 years: 34% 31–44 years old: 33% ≥ 45 years: 33%	20/80	Thoroughbred horse farms	• Established safety practices and horse knowledge are vital to reduce injury when working with Thoroughbreds
Australia (2019) [27]	Retrospective (6 months)	70	37 (IQR 24–52)	27/73	On farms	• 55% was horse trauma • Most large animal injuries were potentially or imminently life-threatening at ED presentation
The Netherlands (2019) [28]	Retrospective (5 years)	945	22 (IQR 3–94)	81.2/18.8	All activities	• Fall commonest MOI for mounted equestrians. Being kicked or trapped was common for unmounted equestrians with a high rate of facial injuries
Single-center studies						
Germany (2023) [29]	Retrospective (3 years)	95	35.3 (range 6–71)	93.7/6.3	All activities	• Accidents caused by holding the reins may result in serious injuries to the hand with 30% requiring an amputation
UK (2023) [30]	Retrospective (7 years)	301	42.7 (16.5, range 4–84)	70.8/29.2	All activities	• Limb injuries are commonest which contrasts the 1970s where head injury prevailed likely due to use of helmets • Better limb safety equipment may prevent injury
USA (2023) [31]	Retrospective (5 years)	143	49.2 (15.5)	62.2/37.8	All activities	• Crush injuries had a high rate of rib fractures • Kicks led to solid organ and facial injuries and falls led to rib fractures and extremity trauma
Australia (2023) [32]	Retrospective (4 years)	809	34 (0–91)	62.5/37.5	All activities	• Horse trauma (81%) was more frequent than cattle (19%) • Fall commonest MOI for horse trauma (68%) and resulted in soft tissue injury (55%); upper limb fracture (19%); lower limb fracture (9%)
Germany (2022) [33]	Retrospective (5 years)	71	34.5 (16.6, range 7–78)	57/14	All activities	• Wearing of protective equipment associated with a shorter LOS and lower risk of postoperative complications
Ireland (2022) [34]	Retrospective (2.5 years)	31	35.5 (16.8)	60/40	Amateur equestrians	• Pelvic and acetabular fractures are associated with other injuries and surgery
Switzerland (2022) [35]	Retrospective (22 years)	501	11 (3, range 2–16)	86.4/13.6	All activities	• Orthopedic injuries 60.9% of lesions, with 4 times more upper limb fractures than lower limb fractures • Over 50% required admission, and almost half had surgery • Head injuries frequent
Scotland (2021) [36]	Retrospective (6 years)	162	37 (18.5)	74.7/25.3	All activities	• Crush or kick resulted in more abdominal visceral injuries and ICU admissions • 5% sent to a major trauma center and 30-day mortality was 0.6%
Finland (2021) [37]	Retrospective (6 years)	39	33.8	92.3/7.7	All activities	• Types of craniofacial fractures: isolated facial (84.6%); isolated cranial (7.7%); combined craniofacial (7.7%) • Surgery rates 48.7% • Severe head and neck injuries (17.9%) with unconsciousness and/or post-traumatic amnesia (41%)
Italy (2019) [38]	Retrospective (72 years)	224	NA	NA	Horse racing (the “Palio,” the oldest race in Italy)	• In 96.1% of the races there was at least one fall and in 28.6% of the races 50% or more of the jockeys fell. In 43.4% of falls, the jockey was taken to ED
Israel (2019) [39]	Retrospective (11 years)	53	11.3 (4.72)	21/79	All activities	• Commonest MOI was fall (58%). Skeletal injuries occurred in 60%, then head (30%), then face (23%). Severe trauma (ISS > 15) was at 28% with 23% admitted to ICU, and 45% required surgery. The mean LOS was 4.3 days • Young boys are at highest risk
Australia (2019) [40]	Retrospective (6 years)	28	31 (IQR 16–76)	57/43	All activities	• For maxillofacial trauma, kick while unmounted was commonest MOI and resulted in many injuries
Germany (2019) [41]	Prospective (1 year)	233	15.59 (5.31, range 6–48)	94/6	Vaulting	• Commonest injuries to lower and upper limbs with bruising and muscle strain • Injury risk increased with greater age, number of falls, greater competitive level, number of events, and previous injuries
USA (2019) [6]	Retrospective (4 years)	222	38.5 (range 4–79)	161/61	All activities	• Older patients (> 54 years) and MOI are predictors of ISS, injury region, health costs, and LOS
USA (2019) [42]	Retrospective (4 years)	27	51 (17)	52/48	All activities	• More serious morbidity in those > 60 years
USA (2019) [43]	Retrospective (16 years)	1195	43 (19)	100% males	Saddle Horn Injuries	• Urethral injury and pubic symphysis diastasis were higher with saddle horn injury than other mechanisms of pelvic ring disruption
Ireland (2018) [44]	Retrospective (1 year)	149	27 (range 5–77)	58/42	All activities	• Recreational equestrians more likely for fracture than professional jockeys, and more likely for surgery • Jockeys experience and training limits injury
Australia (2018) [45]	Retrospective (20 years)	505	12 (IQR 0–15)	80/20	All activities	• Unmounted patients differ from mounted patients in gender, age, use of protective equipment, severity of injuries, and need for ICU

*NA*, not available; *ED*, Emergency Department; *MOI*, mechanism of injury; *ISS*, Injury Severity Score [46]; *ICU*, Intensive Care Unit; *LOS*, length of stay (in-hospital)

### Incidence of injury

It needs to be noted that incidence rates of injury vary geographically and is dependent on the type of horse activity being undertaken. The type of data available and the methods of data collection also influence consistent reporting. Despite this, four studies reported a decrease in horse trauma over their study periods [19, 21, 22, 36], one reported no change [39], and Jones et al. [7] reported a slight non-significant annual increase. A recent assessment of fatal incidents on Australian farms involving children (2001–2019) found that horses were the agent of injury in 5.4% of cases [47]. A retrospective analysis of data from the USA’s Nationwide Emergency Department Sample identifying horse trauma-related visits revealed 21,899 visits but when weighted these represented 100,964 visits or 0.64 per 10,000 persons (95% CI, 0.60–0.68) [1].

### Characteristics of injured equestrians

#### Gender

Of the 39 studies included in the review, 20 reported that horse-related trauma most frequently involved females with the disparity ranging between 52 [42] and 93.7% [29]. This skewing is usually explained by the fact that many recreational and amateur horse-related activities are predominantly undertaken by females [9] with British Equestrian recently reporting a female participation rate of 74% [2]. However, Gross et al. [39] found males outnumbered females in a pediatric population. In a study by Samuels et al. [14] that undertook subgroup analyses, it was found occupational injuries affected predominantly males between the ages of 18 and 49 years, while non-occupational injuries predominantly affected females less than 18 years old and greater than 60 years old (*p* < 0.001). Where the type of horse- activity is specified such as in a competition it was found that the frequency of injury did not differ significantly in male and female participants [23, 41].

#### Age

In respect to age, some studies reported mean age whereas others reported median. The years of age between 22 and 49 is the most common with females generally in their late-20 s to mid-30 s and males in their early 40 s. However, two large national epidemiological studies in the USA found younger populations were more predominant. Asa et al. [1] who analyzed horse trauma from the Nationwide Emergency Department Sample (2010–2014; *N* = 21,899) found the proportion of visits were highest in females aged 15–19 years. Similarly, Acton et al. [19] using the National Electronic Injury Surveillance System (1990 – 2017; *N* = 1,836,536) found the injury rate per 100,000 population was highest among 5–18-year-olds (gender not specified). Studies that concentrated on pediatrics have a mean age of 11 years with male children tending to be younger [30, 35, 39].

#### Occupational or non-occupational

Abdulkarim et al. [44] reported that 82% of those injured were recreational horse equestrians. Whereas Samuels et al. [14] established that 6.1% of injuries were occupation-related and furthermore, of the non-occupational injuries, 43.7% occurred on a farm, 8.6% occurred in sporting locations, 21.7% occurred in recreational locations, and 20% occurred in residential locations.

### Characteristics of horse trauma

#### Mechanism of injury

The most common mechanism of injury was a fall from a horse, with the proportion ranging from 45.1 to 86.4% of all mechanisms [36, 41]. Equestrians who had fallen had the highest injury scores, were most likely to be admitted to hospital, had the most diagnostic imaging performed, and tied with horse kick injuries for the longest mean hospital stay [6]. Kicks, usually while unmounted, accounted for around 23% of injuries [29] and often caused facial fractures [33, 40]. Other important mechanisms included trampling (10%) as well as strikes, bites, and being squashed [32]. Injuries also occur in relation to being caught up in equipment such reins, leads, and stirrups or landing on saddle horns [29, 35, 43]. Two studies reported that approximately 20% of injured equestrians had a combined mechanism such as being trampled or knocked following a fall [7, 28].

#### Injured body regions and types of injury

With horse trauma, the musculoskeletal system is commonly affected. Although dependent on specific study categorizations, generally in order, the most frequently injured body parts were the thorax, upper extremities and lower extremities, spinal column, head, face, and abdomen [14, 16, 28]. Table 2 summarizes the injury patterns by body part reported in the four largest epidemiological studies identified for this review. It is important to point out that in over 28% of the injured cases, the equestrian suffered multiple injuries resulting from the same injury event [4].

**Table 2:** Injury patterns (%) by body part reported in four largest epidemiological studies

Study	Head/neck	Thorax	Upper extremity	Lower extremity
Asa et al. [1]	22.8	30	25	20
Mutore et al. [16]	23	37	26 (combined)	
Acton et al. [19]	22.6	29	29	18
Meredith et al. [4]	34	27	29	23

Most diagnosed conditions were contusions (41.8%), followed by one or more fractures (39%), traumatic brain injury (13%), and visceral organ injury (3.1%) [1, 4, 17, 28, 44]. Most thoracic injuries were caused by fall from horse (78.9%) [7]. Dick et al. [36] found that there was an associated flail segment in 16% of patients with a rib fracture. Of patients with a pneumothorax, 40% were complicated by a hemothorax and 8.33% by tension. Injury to the upper limbs (27%) occurred primarily to the shoulder, scapula, clavicle, and hand [7, 17]. For the lower limbs (20.3%), tibia and fibula fractures were most common [1, 35].

Head injuries accounted for about 22% of the injuries [19], with about half of these injuries being traumatic brain injuries (TBI) [1]. According to Jones et al. [7], nearly 14% of all TBIs had a serious or severe Abbreviated Injury Scale (AIS) [48] score of 3–4. TBI events by mechanism were as follows: fall from horse (79.5%), kicked (10.2%), and knocked (4.5%). When skull and jaw fractures were diagnosed, these were found to occur at the vault of the skull, base of the skull, mandible, orbit and zygoma, and maxilla and nose [36]. The commonest head injury sustained was concussion with complex head injuries represented by subarachnoid (2.5%) and subdural hematomas and temporal lobe contusion (0.62%) [36].

Puolakkainen et al. [37] in a study of craniofacial fractures (*n* = 39) reported that over 90% of the patients sustained a facial fracture. Furthermore, Singleton et al. [40] found that out of a total of 61 facial fractures (those that did and did not require operative treatment), 54 were treated operatively. Of them, 44 were managed with internal fixation, nine with closed reduction, and one with a combination of both. When dentoalveolar and soft-tissue lacerations were included, there were 83 injuries in total with the most common fractures being naso-orbitoethmoidal and orbital fractures. Of note was that over a quarter of injuries to the face (26.9%), resulting from kicks [7]. Kicks or crushing were also responsible for abdominal visceral injuries seen in around 14% of reported cases, often resulting in liver, splenic, kidney laceration, and/or pancreatic contusion [7, 16, 36].

With the pediatric population, head trauma (30%) was the most common injury, followed by injuries of the upper and lower extremities (28%). Other injuries included the face (23%), chest (13%), abdomen (8%), and spine (4%) [4, 35, 39, 45]. The higher rate of head trauma was put down to children having a greater head-to-body size ratio and thinner skull bones [49, 50]. Of note, Wolyncewicz et al. [45] found that unmounted children suffered more severe injuries than mounted children.

Injury patterns also vary with the type of horse activity. For example, in vaulting which is a combination of dancing and gymnastics on horseback [41], the most frequently injured body part was the lower extremity (45.6%), followed by the upper extremity (27.2%), torso (21.6%), and head (5.6%) and rather than fractures and dislocations, the most common injury types were contusions (46.3%) followed by ligament injuries (28.9%) [4]. In the horse racing industry, jockeys mostly experience bruises and abrasions followed by fractures and dislocations of the upper and lower limbs; however, fracture and concussion incidences were higher in amateur than professional jockeys [21, 38]. Whereas in grooms, when working unmounted, the most common injuries reported were bruises (23.47%), lower back pain (14.5%), muscle strain (13.5%), upper back/neck pain (8.7%), lacerations (5.97%), tendon/ligament damage (5.2%), and suspected concussion (5.1%) [15]. In horse activities such as rodeos, an equestrian may be bucked into the air and subsequently land with their perineum striking a rigid saddle horn with consequential lower genitourinary injuries particularly in those individuals who fractured their pelvis due to saddle horn injury [43].

#### Hospital admission, length of stay, and rehabilitation

Most individuals with horse trauma (85.7–87.3%) were treated in the Emergency Department (ED) and discharged with the rest admitted [1, 19]. Of patients admitted, reports of those requiring surgery varied between 29.1 [28] and 55.6% [29]. Examples of the various surgical procedures carried out in a pediatric population included closed reduction of limb fractures (28.5%), closed reductions requiring percutaneous stabilization (31.5%), open limb fractures requiring reduction and fixation (18.5%), and spinal fixations, craniotomies, facial repairs, laparotomy, wound explorations and sutures (13.9%) [35]. Other reports indicated that between 18 and 27% of patients required Intensive Care Unit (ICU) admission [18, 28, 30] and furthermore Buchanan et al. [18] reported that 276 (5%) of patients in their study had mechanical ventilation. Of note, Acton et al. [19] found that mounted injuries were 2.10 (95% CI, 1.59–2.77) times more likely to be admitted than unmounted. Older patients, < 60 years of age (RR, 2.41; 95% CI, 2.01–2.90), were more likely to be hospitalized than younger patients. Patients with a fracture (RR, 3.22; 95% CI, 2.89–3.60) or a concussion/closed head injury (RR, 2.00; 95% CI, 1.79–2.23) were more likely to be admitted than patients with other injuries. Patients injured from a horse bucking, rearing, or spooking were 1.55 (95% CI, 1.36–1.78) times more likely to be admitted than patients injured from other mechanisms [19].

Regarding length of stay (LOS) in hospital, most studies placed in the 3- to 4-day band (range 0–15) [14, 16, 17, 31, 35, 39, 40] with more severe injuries (ISS equal/greater than 4) requiring 8.56 (± 10.84, range 1–64) days [30]. Older patients had longer LOS although not recording any worse injuries, the explanation being perhaps the case of more cautious monitoring [33, 42].

Almost 50% of all cases required professional rehabilitation [13] with O'Connor et al. [17] reporting that 39.13% of jockeys self-rehabilitated and 8.70% completed no rehabilitation. Aref-Ali Gharooni et al. [30] administered the Glasgow Outcome Scale [51] to patients on discharge from a major trauma center in the UK with nearly 75% with no disability, 22.3% with moderate disability (i.e., minor deficits that do not affect function), 1.3% with severe disability, and 0.7% in a persistent vegetative state. Given the importance of mental health, one study [12] conducted a cross-sectional survey (*N* = 511) to evaluate concussion history, depression symptoms, resilience, and well-being in equestrian athletes. Almost a third of the sample had depression, and there were significant relationships between a history of concussion, depression, and a sense of low well-being.

#### Severity of injury

##### GCS

In studies that reported on the Glasgow Coma Scale (GCS) [52], around 88% of individuals presented with scores between 13 and 15 [16, 18, 30, 31, 44]. Furthermore, Mutore et al. [16] found severe neurological impairment classified as a GCS score of 3–8 on presentation was observed in 3.58% patients. Within the severe impairment group, head and neck injuries were the most likely cause. Puolakkainen et al. [37] found that patients with GCS score < 12 were more likely to have cranial fractures (*p* = 0.002).

##### ISS/SBP

Reports regarding Injury Severity Score (ISS) [46] have shown varying results and are most likely due to such factors as different mechanisms, cohorts, and types of riding. For example, the mean ISS ranges from 5 [7] to 11 (SD 6.4) [31], median 4 (IQR = 1) [7] to 12 (range 4–45) [30] with similar fluctuations for the pediatric studies [35, 39, 45]. Both Van Balen, Barten [28] and Gross, Hadar [39] reported on cases with an ISS greater than 15. Two studies noted systolic blood pressure (SBP) as a measure for hemodynamic shock [16, 18]. Approximately 95% of Emergency Department (ED) presentations (*N* = 23619) had a SBP of ≥ 90 mm Hg with 1.40% of patients meeting the threshold of an SBP < 90 mm Hg whereas Buchanan et al. [18] reported a mean (SD) SBP of 132 (22) in 5731 patients with 1.2% SBP < 90.

#### Mortality

Mortality rates are noted as between 0.6 and 1.0% [30, 36]. The most common critical injuries were traumatic brain injuries (74.83%), thoracic injuries (18.44%), abdominal injuries (5.31%), and 2.19% patients dying to extremity injuries [16]. Lethal head injuries often follow falls and kicks [26]. Higher ISS scores resulted in the more likelihood of death [16]. Meredith et al. [4] found that there was an increase of 5.1% in the odds of fatality for every year increase in age of the patient with Byard [26] noting an age range of 8–73 years (mean 47 years) and as with Meredith et al. [4] a male–female ratio of approximately 5:3. Helmets and other protective equipment such as vests, boots, and safety stirrups have been shown to decrease traumatic injury with many horse-riding associations promoting the use of such items [18].

#### Use and effects of protective equipment

Helmets and other protective equipment such as vests, boots, and safety stirrups have been shown to decrease traumatic injury with many horse-riding associations promoting the use of such items [13]. In relation to the use and effects of protective equipment, Samuels and Bettis [14] (*n* = 3911) reported the use of helmets as low (19.8% helmet worn; 80.2% no helmet worn) and showed that the use of helmets significantly reduced the incidence of a head injury (AIS > 2) among equestrians (*p* = 0.003). Similarly, an examination of head injuries in children (*n* = 505) [45] found that equestrians not wearing a helmet were 12 times more likely to sustain a major head injury (AIS 4–5) than those who were wearing a helmet (OR 11.6: 95% CI 3.8, 36.9). Stier and Tavassol [33] in a study of maxillofacial fractures (*n* = 71) found that helmets were worn by 67% of beginner equestrians, but only by 44% of advanced equestrians and 33% of professional equestrians despite most injuries occurring in the latter two groups. The findings of these recent studies are consistent with previous ones that the wearing of helmets is associated with a reduction in head injuries, but the rates of use remain low [26].

## Discussion

The purpose of this review was to critically appraise and summarize reports on horse trauma and to examine the characteristics of injured equestrians, characteristics of trauma, and clinical outcomes since 2018. Previous reviews were before this time frame. Meredith et al. [8] examined equestrian accidents from 1973 to 2017, and Havlik [9] reviewed equestrian sport-related injuries (2007–2009) while Gates and Lin [11] examined head and spinal injuries specifically (2009–2019) and Zuckerman et al. [10] focused on functional and structural traumatic brain injury in equestrian sports which included studies from 1993 to 2013. Therefore, a comprehensive review of this topic is timely.

In relation to characteristics of injured equestrians, much of the injured population were females with some reports up to 90%, and most equestrians were teenagers to younger adults up to around 40 years of age. This is like that reported in previous reviews [8–11]. Therefore, the demographics of the population under study has not changed over this period and this result is put down to the fact that there continues to be a high participation rate of females in equestrian-related activities [2, 3]. As with others, recreational horse activities mostly involve females [9], whereas men get injured while working [11]. Interestingly, in high-level competitions, there is no difference in gender which may indicate that the levels of skill and experience of the equestrian and the training of the horse are risk factors for horse trauma and support the need for more comprehensive and standardized data collection regarding equestrian and horse characteristics and mechanism of injury [23, 41].

In regard to characteristics of horse trauma, little has changed over the decades with most common mechanism of injury while mounted being a fall from the horse whereas in unmounted situations it was a kick from the horse [8–11]. There is also a reasonable possibility that an equestrian will have a combined mechanism such as being trampled following a fall. As with the review by Havlik [9], we found the most frequently injured body regions were the thorax and upper extremities, followed by the head and then lower extremities. However, Meredith et al. [8] found a greater predominance of head injuries and particularly noted higher occurrences with pediatric populations which is also supported by Gross et al. [39] who examined characteristics of horse-related injuries in Israeli children (*n* = 53). These findings infer again that the level of skill and experience of the equestrian are potential risk factors for horse trauma. While difficult to quantify, it is postulated that awareness of the horse and the surroundings, which comes with knowledge and experience, may reduce the incidence of injury [13, 44]. It is not evident from this review whether a horse safety awareness program for equestrians would reduce the incidence of mounted and unmounted injury.

In line with the most common mechanism of fall which can occur from a height of 9 feet/2.7 m/18 hands and at times at speeds of 40 miles/64 km/h, the most frequently diagnosed injuries were skin and/or bone contusion, fractures, head and neck injury, and visceral organ injury which in general correspond to previous reviews but with the proviso that data collection and analysis remain inconsistent [9]. Horses also weigh up to 1200 pounds/545 kg and can deliver 1000 newtons of force from a single kick [9] making injuries, even if the individual is unmounted, significant and common to the face, lower extremity, and abdomen. Given the variable size, speed, height, training, and temperament of different breeds of horse and the obvious impact this can have on severity and pattern of injury, it is important to gather this information at the time of presentation to hospital. The less frequent injury to the perineum and lower genitourinary tract sustained due to saddle horn strike is an injury that can have significant effect on morbidity in terms of fertility, sexual function, and cosmesis and is something that has not been commented on in the literature. As some of the mishaps occur because of the unpredictable nature of this large animal, it does not matter if the environment is recreational or a workplace [14, 44]; it is recommended that protective equipment, such as helmets and protective vests, be used across the board to control the incidence and severity of injury [8, 10]. It has been established that the incidence of severe head injury is lower in groups that wear helmets [14, 31, 33].

Other reviews have not reported on severity of injuries [8, 9, 11] and although there were reports of serious injuries across our reviewed studies, the average patient with horse trauma was not severely injured with medians of ISS reported as 4 (IQR = 1) [7] to 12 (range 4–45) [30]. Additionally, Mutore et al. [16] recorded systolic blood pressure (SBP) as a measure for hemodynamic shock with 1.40% of individuals meeting its threshold of < 90 mm Hg. GCS scores in most studies were noted as between 13 and 15 but if head and neck injuries were involved these usually dropped below 12 [16, 37]. Given the potential for severe injury resulting in significant morbidity and mortality, horse trauma should not be underestimated and should be treated as seriously as other mechanisms such as fall from height, motor vehicle, or motorbike accident. Inclusion of vital signs and GCS should be a part of a standardized horse trauma protocol.

Considering the data in relation to severity of injury, it is no surprise that well over 80% of individuals are treated in the ED and then released [19]. However, if admission is required, surgery for fractures and organ damage may be part of the course, through to ICU admission. Length of stays, and if required, rehabilitation, varies according to the injury with the resultant health system costs and if the case, cost for workplaces, and insurance companies. The rate of hospitalization has not changed, and this should be the driver for more injury prevention strategies to be put in place. Most of the research concentrated on physical injuries but mental health concerns particularly depression following concussion need greater attention for equestrians particularly in relation to getting treatment and being involved in health promotion programs [12]. Fortunately, death continues to occur at minimal rate with Meredith et al. [8] citing it as 0.17 per 100,000 people in a 1993 New Zealand study.

Since there has been little change in how data is collected for horse trauma over the last few decades, we would recommend a standardized approach for data collection with the intention of prospective design to ensure complete data collection, for example, to include if the equestrian was using protective equipment, the level of experience of the equestrian, environmental conditions, and importantly the characteristics of the horse [53]. More work could also take place on the long-term effects of horse trauma, for example, head injury given the recent focus of other sports on chronic traumatic encephalopathy or fertility given the seriousness of lower genitourinary tract injury sustained from saddle horn injury.

## Conclusion

Horse trauma remains an ongoing presentation for health services. Since 2018, little has changed in relation to outcomes except for a continued decline in head injuries due to the use of protective equipment. A high risk of significant injury remains so there is a continuing need for more work to be done in this space. This review demonstrated that this is a global issue and there is potential to standardize data collection across these jurisdictions and work with the different types of horse-related industries at the recreational and occupational level to achieve better outcomes for equestrians and health services.
